# 2-[2-(2-Pyrid­yl)eth­yl]isoindolinium perchlorate

**DOI:** 10.1107/S1600536809032024

**Published:** 2009-08-19

**Authors:** Ray J. Butcher, Yohannes T. Tesema, Teshome B. Yisgedu, Yilma Gultneh

**Affiliations:** a Department of Chemistry, Howard University, 525 College Street NW, Washington, DC 20059, USA

## Abstract

In the title salt, C_15_H_17_N_2_^+^·ClO_4_^−^, the isoindoline N atom is protonated and an intra­molecular N—H⋯N hydrogen bond occurs. In the crystal, N—H⋯O and numerous weak C—H⋯O inter­actions occur between the cation and anion. The O atoms of the perchlorate anion are disordered over four sets of sites with occupancies of 0.438 (4), 0.270 (9), 0.155 (8) and 0.138 (5).

## Related literature

For further information on the synthesis, see: Bonnett *et al.* (1983 ▶); Meyers & Santi­ago (1995 ▶).
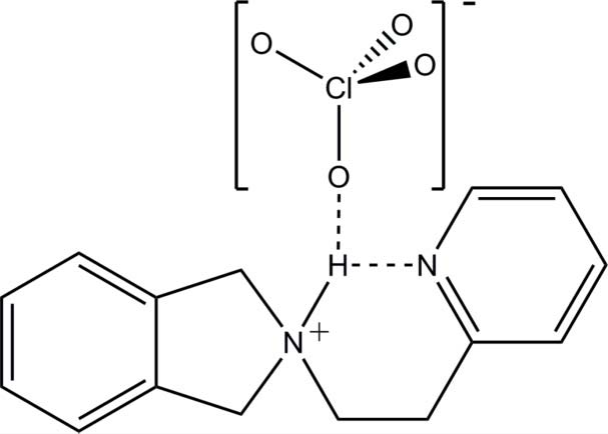


## Experimental

### 

#### Crystal data

C_15_H_17_N_2_^+^·ClO_4_^−^
*M* *_r_* = 324.76
Orthorhombic, 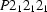

*a* = 7.7941 (9) Å
*b* = 11.7032 (19) Å
*c* = 16.850 (3) Å
*V* = 1537.0 (4) Å^3^
*Z* = 4
Mo *K*α radiation
μ = 0.27 mm^−1^
*T* = 293 K
0.5 × 0.2 × 0.08 mm

#### Data collection

Bruker P4 diffractometer
Absorption correction: ψ scan (North *et al.*, 1968 ▶) *T*_min_ = 0.241, *T*_max_ = 0.265 (expected range = 0.890–0.979)
2027 measured reflections
2001 independent reflections
1660 reflections with *I* > 2σ(*I*)
*R*_int_ = 0.025
3 standard reflections every 97 reflections intensity decay: <2%

#### Refinement

*R*[*F* ^2^ > 2σ(*F* ^2^)] = 0.044
*wR*(*F* ^2^) = 0.120
*S* = 1.03
2001 reflections
329 parameters
245 restraints
H-atom parameters constrained
Δρ_max_ = 0.21 e Å^−3^
Δρ_min_ = −0.15 e Å^−3^

### 

Data collection: *XSCANS* (Bruker, 1997 ▶); cell refinement: *XSCANS*; data reduction: *SHELXTL* (Sheldrick, 2008 ▶); program(s) used to solve structure: *SHELXS97* (Sheldrick, 2008 ▶); program(s) used to refine structure: *SHELXL97* (Sheldrick, 2008 ▶); molecular graphics: *SHELXTL*; software used to prepare material for publication: *SHELXTL*.

## Supplementary Material

Crystal structure: contains datablocks I, global. DOI: 10.1107/S1600536809032024/hb5043sup1.cif

Structure factors: contains datablocks I. DOI: 10.1107/S1600536809032024/hb5043Isup2.hkl

Additional supplementary materials:  crystallographic information; 3D view; checkCIF report

## Figures and Tables

**Table 1:** Hydrogen-bond geometry (Å, °)

*D*—H⋯*A*	*D*—H	H⋯*A*	*D*⋯*A*	*D*—H⋯*A*
N2—H2*B*⋯N1	0.91	2.11	2.769 (4)	129
N2—H2*B*⋯O4	0.91	2.39	3.197 (9)	148
N2—H2*B*⋯O2*A*	0.91	2.53	3.222 (19)	133
N2—H2*B*⋯O1*C*	0.91	2.59	3.390 (16)	147
C2—H2*A*⋯O1*B*^i^	0.93	2.30	3.062 (11)	139
C3—H3*A*⋯O1*A*^ii^	0.93	2.62	3.193 (10)	121
C7—H7*B*⋯O2*A*^iii^	0.97	2.50	3.232 (16)	132
C8—H8*A*⋯O3	0.97	2.55	3.383 (10)	144
C8—H8*A*⋯O2*B*	0.97	2.39	3.168 (19)	136
C8—H8*A*⋯O3*C*	0.97	2.29	3.189 (17)	154
C8—H8*B*⋯O4*B*^iv^	0.97	2.48	3.167 (17)	128
C11—H11*A*⋯O3*A*^v^	0.93	2.55	3.27 (2)	135
C11—H11*A*⋯O3*B*^v^	0.93	2.57	3.47 (2)	162
C13—H13*A*⋯O3^vi^	0.93	2.48	3.299 (9)	148
C13—H13*A*⋯O4*A*^vi^	0.93	2.20	2.976 (8)	140
C13—H13*A*⋯O1*B*^vi^	0.93	2.56	3.44 (2)	158
C13—H13*A*⋯O3*C*^vi^	0.93	2.70	3.444 (17)	138
C15—H15*A*⋯O1^iii^	0.97	2.55	3.423 (9)	150
C15—H15*B*⋯O2^vi^	0.97	2.48	3.425 (10)	163
C15—H15*B*⋯O4*A*^vi^	0.97	2.55	3.194 (19)	124

Symmetry codes: (i) 
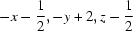
; (ii) 
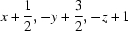
; (iii) 
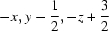
; (iv) 
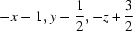
; (v) 
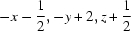
; (vi) 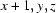.

**Fig. 1.**
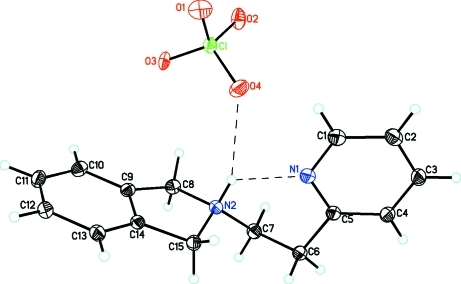
The title compound showing the N—H···N bond (dashed bonds). Ellipsoids are drawn at the 20% probabilty level.

**Fig. 2.**
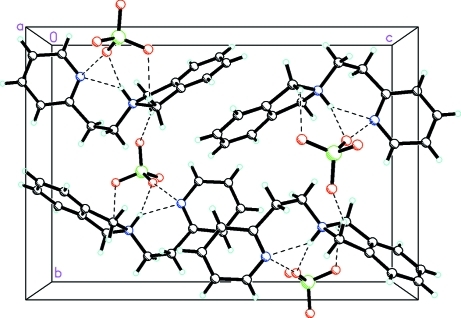
The packing arrangement viewed down the a axis showing the N—H···O and intermolecular C—H···O interactions (dashed bonds).

## supplementary crystallographic information

### Comment

The stucture of the title compound, (I), is shown below. Dimensions are available in the archived CIF.

The crystal structure shows that the N atom of 2,3-dihydro-1*H*-isoindoline is protonated and involved in hydrogen bonding to the nearest pyridyl N atom (Table 1). The perchlorate ion O atoms are also involved in the hydrogen bonding with the isoindoline H atom. The amine H atom is also involved in hydrogen bonding to the perchlorate O atoms and there are extensive but weak interionic C—H···O interactions between the cation and anion (Table 1).

### Experimental

The neutral ligand 2-(2-pyridylethyl)-2,3-dihydro-1*H*-isoindole, was synthesized by reacting 2-(2-aminoethyl)pyridine and α,α'-dibromo-*o*-xylene in THF in the presence of triethylamine as a base and purified by column chromatography using modified literature methods of Bonnett *et al.* (1983) & Meyers *et al.* (1995). The perchlorate salt of the protonated ligand (1) was formed by addition of one equivalent of an aqueous solution of HClO_4_ to a solution of the neutral ligand in methanol and allowing the resulting solution to stand yielded pale pink needles of (I).

### Refinement

The perchlorate anion is disordered over four conformations with occupancy factors of 0.438 (4), 0.270 (9), 0.155 (8), and 0.138 (5). These were constrained to adopt a tetrahedral geometry. This disorder and its modelling resulted in 245 restraints. The H atoms were idealized with an N—H distance of 0.91Å and C—H distances were idealized at 0.93 (aromatic C—H), 0.96 (CH_3_), and 0.97 (CH_2_) Å and *U*_iso_(H) = 1.2*U*_eq_(C) (1.5*U*_eq_(C) for the CH_3_ protons).  Since a unique data set was collected there were no Friedel pairs and no attempt was made to establish the absolute sturcture.

### Crystal data

**Table:**

C_15_H_17_N_2_^+^·ClO_4_^−^	*F*(000) = 680
*M**_r_* = 324.76	*D*_x_ = 1.403 Mg m^−^^3^
Orthorhombic, *P*2_1_2_1_2_1_	Mo *K*α radiation, λ = 0.71073 Å
Hall symbol: P 2ac 2ab	Cell parameters from 39 reflections
*a* = 7.7941 (9) Å	θ = 5.1–12.5°
*b* = 11.7032 (19) Å	µ = 0.27 mm^−^^1^
*c* = 16.850 (3) Å	*T* = 293 K
*V* = 1537.0 (4) Å^3^	Needle, pale pink
*Z* = 4	0.5 × 0.2 × 0.08 mm

### Data collection

**Table:**

Bruker P4 diffractometer	1660 reflections with *I* > 2σ(*I*)
Radiation source: fine-focus sealed tube	*R*_int_ = 0.025
graphite	θ_max_ = 27.5°, θ_min_ = 2.4°
ω scans	*h* = 0→10
Absorption correction: ψ scan (North *et al.*, 1968)	*k* = 0→15
*T*_min_ = 0.241, *T*_max_ = 0.265	*l* = 0→21
2027 measured reflections	3 standard reflections every 97 reflections
2001 independent reflections	intensity decay: <2

### Refinement

**Table:**

Refinement on *F*^2^	Primary atom site location: structure-invariant direct methods
Least-squares matrix: full	Secondary atom site location: difference Fourier map
*R*[*F*^2^ > 2σ(*F*^2^)] = 0.044	Hydrogen site location: inferred from neighbouring sites
*wR*(*F*^2^) = 0.120	H-atom parameters constrained
*S* = 1.03	*w* = 1/[σ^2^(*F*_o_^2^) + (0.0626*P*)^2^ + 0.2989*P*] where *P* = (*F*_o_^2^ + 2*F*_c_^2^)/3
2001 reflections	(Δ/σ)_max_ = 0.005
329 parameters	Δρ_max_ = 0.21 e Å^−^^3^
245 restraints	Δρ_min_ = −0.15 e Å^−^^3^

### Special details

**Table:**

Geometry. All e.s.d.'s (except the e.s.d. in the dihedral angle between two l.s. planes) are estimated using the full covariance matrix. The cell e.s.d.'s are taken into account individually in the estimation of e.s.d.'s in distances, angles and torsion angles; correlations between e.s.d.'s in cell parameters are only used when they are defined by crystal symmetry. An approximate (isotropic) treatment of cell e.s.d.'s is used for estimating e.s.d.'s involving l.s. planes.
Refinement. Refinement of *F*^2^ against ALL reflections. The weighted *R*-factor *wR* and goodness of fit *S* are based on *F*^2^, conventional *R*-factors *R* are based on *F*, with *F* set to zero for negative *F*^2^. The threshold expression of *F*^2^ > σ(*F*^2^) is used only for calculating *R*-factors(gt) *etc*. and is not relevant to the choice of reflections for refinement. *R*-factors based on *F*^2^ are statistically about twice as large as those based on *F*, and *R*- factors based on ALL data will be even larger.

### Fractional atomic coordinates and isotropic or equivalent isotropic displacement parameters (Å^2^)

**Table:**

	*x*	*y*	*z*	*U*_iso_*/*U*_eq_	Occ. (<1)
Cl	−0.45920 (11)	0.96522 (8)	0.73473 (5)	0.0643 (3)	
O1	−0.4598 (13)	1.0879 (4)	0.7432 (7)	0.093 (3)	0.438 (4)
O2	−0.5971 (10)	0.9347 (9)	0.6824 (5)	0.101 (3)	0.438 (4)
O3	−0.4861 (13)	0.9137 (8)	0.8101 (3)	0.074 (2)	0.438 (4)
O4	−0.3007 (9)	0.9305 (9)	0.7017 (6)	0.105 (3)	0.438 (4)
O1A	−0.497 (3)	0.8798 (16)	0.6772 (10)	0.099 (5)	0.155 (8)
O2A	−0.2811 (10)	0.9589 (19)	0.7554 (14)	0.100 (6)	0.155 (8)
O3A	−0.494 (3)	1.0759 (10)	0.7025 (15)	0.103 (5)	0.155 (8)
O4A	−0.561 (3)	0.948 (2)	0.8038 (8)	0.104 (5)	0.155 (8)
O1B	−0.466 (3)	0.9618 (18)	0.8191 (3)	0.100 (6)	0.138 (5)
O2B	−0.404 (3)	0.8561 (9)	0.7054 (11)	0.099 (5)	0.138 (5)
O3B	−0.341 (2)	1.0506 (14)	0.7094 (12)	0.100 (4)	0.138 (5)
O4B	−0.6256 (14)	0.9896 (18)	0.7036 (13)	0.104 (5)	0.138 (5)
O1C	−0.2804 (8)	0.9697 (14)	0.7176 (9)	0.088 (4)	0.270 (9)
O2C	−0.5525 (18)	0.9411 (15)	0.6637 (6)	0.111 (5)	0.270 (9)
O3C	−0.492 (2)	0.8776 (12)	0.7918 (8)	0.096 (4)	0.270 (9)
O4C	−0.5146 (19)	1.0728 (8)	0.7659 (10)	0.111 (4)	0.270 (9)
N1	−0.0054 (4)	0.8416 (2)	0.61178 (17)	0.0633 (7)	
N2	−0.0414 (3)	0.7346 (2)	0.75736 (15)	0.0546 (6)	
H2B	−0.0805	0.7936	0.7272	0.065 (10)*	
C1	−0.0438 (5)	0.9290 (3)	0.5639 (2)	0.0716 (9)	
H1A	−0.0860	0.9961	0.5861	0.084 (13)*	
C2	−0.0232 (5)	0.9233 (4)	0.4823 (2)	0.0762 (10)	
H2A	−0.0468	0.9865	0.4507	0.114 (16)*	
C3	0.0328 (5)	0.8230 (4)	0.4489 (2)	0.0798 (11)	
H3A	0.0460	0.8167	0.3943	0.077 (11)*	
C4	0.0691 (5)	0.7319 (4)	0.4976 (2)	0.0702 (9)	
H4A	0.1059	0.6629	0.4762	0.086 (13)*	
C5	0.0502 (5)	0.7442 (3)	0.57909 (19)	0.0609 (8)	
C6	0.0970 (5)	0.6493 (3)	0.6367 (2)	0.0677 (9)	
H6A	0.2089	0.6658	0.6591	0.083 (13)*	
H6B	0.1062	0.5782	0.6074	0.073 (11)*	
C7	−0.0297 (5)	0.6324 (3)	0.7044 (2)	0.0652 (8)	
H7A	−0.1422	0.6163	0.6825	0.069 (10)*	
H7B	0.0053	0.5668	0.7356	0.080 (11)*	
C8	−0.1673 (4)	0.7187 (3)	0.8247 (2)	0.0626 (8)	
H8A	−0.2813	0.7439	0.8098	0.105 (15)*	
H8B	−0.1724	0.6395	0.8414	0.086 (12)*	
C9	−0.0935 (4)	0.7931 (3)	0.88894 (19)	0.0552 (7)	
C10	−0.1673 (5)	0.8278 (3)	0.9602 (2)	0.0690 (9)	
H10A	−0.2794	0.8076	0.9729	0.095 (14)*	
C11	−0.0696 (6)	0.8932 (3)	1.0117 (2)	0.0743 (11)	
H11A	−0.1162	0.9156	1.0600	0.069 (10)*	
C12	0.0928 (6)	0.9252 (3)	0.9930 (2)	0.0721 (10)	
H12A	0.1547	0.9704	1.0282	0.112 (16)*	
C13	0.1681 (5)	0.8917 (3)	0.9219 (2)	0.0654 (8)	
H13A	0.2787	0.9146	0.9088	0.124 (19)*	
C14	0.0738 (4)	0.8232 (3)	0.87123 (18)	0.0546 (7)	
C15	0.1273 (4)	0.7717 (3)	0.79305 (19)	0.0612 (8)	
H15A	0.2032	0.7070	0.8009	0.064 (10)*	
H15B	0.1841	0.8277	0.7597	0.079 (12)*	

### Atomic displacement parameters (Å^2^)

**Table:**

	*U*^11^	*U*^22^	*U*^33^	*U*^12^	*U*^13^	*U*^23^
Cl	0.0620 (5)	0.0772 (5)	0.0537 (4)	−0.0053 (5)	0.0037 (4)	−0.0008 (4)
O1	0.083 (6)	0.077 (4)	0.121 (7)	−0.012 (4)	−0.002 (6)	−0.003 (4)
O2	0.094 (6)	0.146 (8)	0.062 (4)	−0.043 (5)	−0.005 (4)	−0.008 (5)
O3	0.082 (5)	0.093 (6)	0.047 (3)	0.015 (5)	0.017 (3)	−0.004 (3)
O4	0.094 (5)	0.110 (7)	0.112 (6)	0.028 (5)	0.040 (5)	0.003 (6)
O1A	0.099 (10)	0.117 (10)	0.082 (8)	−0.002 (9)	−0.010 (8)	−0.028 (8)
O2A	0.084 (9)	0.092 (10)	0.126 (12)	−0.007 (9)	0.028 (10)	0.011 (11)
O3A	0.085 (9)	0.111 (9)	0.112 (11)	0.031 (8)	−0.016 (9)	0.023 (9)
O4A	0.096 (10)	0.133 (11)	0.085 (9)	−0.003 (10)	0.044 (8)	−0.022 (9)
O1B	0.100 (10)	0.121 (12)	0.079 (9)	0.001 (11)	0.015 (9)	−0.017 (9)
O2B	0.099 (10)	0.102 (9)	0.096 (9)	0.012 (9)	0.006 (9)	−0.013 (8)
O3B	0.087 (8)	0.095 (8)	0.120 (9)	−0.014 (8)	0.021 (8)	0.002 (8)
O4B	0.081 (9)	0.128 (10)	0.101 (10)	0.022 (9)	−0.013 (9)	0.000 (9)
O1C	0.078 (6)	0.079 (7)	0.107 (9)	−0.010 (5)	0.056 (6)	−0.025 (7)
O2C	0.123 (10)	0.150 (9)	0.059 (6)	−0.021 (9)	−0.013 (7)	−0.010 (7)
O3C	0.082 (7)	0.107 (9)	0.099 (8)	0.007 (7)	0.015 (7)	0.016 (7)
O4C	0.096 (8)	0.117 (7)	0.120 (8)	0.039 (6)	0.008 (7)	−0.025 (7)
N1	0.0600 (17)	0.0669 (17)	0.0630 (15)	0.0055 (13)	−0.0045 (14)	−0.0003 (13)
N2	0.0552 (14)	0.0559 (13)	0.0526 (13)	0.0009 (12)	−0.0042 (13)	0.0014 (11)
C1	0.066 (2)	0.069 (2)	0.080 (2)	−0.0005 (19)	−0.004 (2)	0.0048 (18)
C2	0.060 (2)	0.091 (3)	0.078 (2)	−0.004 (2)	0.0013 (19)	0.026 (2)
C3	0.0581 (19)	0.122 (3)	0.0588 (19)	0.006 (2)	0.0058 (18)	0.010 (2)
C4	0.061 (2)	0.090 (2)	0.0599 (18)	0.0087 (19)	−0.0008 (17)	−0.0076 (18)
C5	0.0543 (17)	0.0722 (19)	0.0561 (16)	0.0054 (17)	−0.0032 (16)	−0.0035 (15)
C6	0.075 (2)	0.070 (2)	0.0584 (18)	0.0152 (18)	−0.0067 (18)	−0.0095 (16)
C7	0.076 (2)	0.0574 (17)	0.0620 (17)	0.0011 (17)	−0.0066 (19)	−0.0045 (14)
C8	0.0541 (18)	0.0631 (19)	0.071 (2)	−0.0068 (16)	0.0066 (16)	0.0027 (16)
C9	0.0571 (18)	0.0510 (15)	0.0576 (17)	0.0052 (14)	0.0056 (14)	0.0081 (14)
C10	0.070 (2)	0.065 (2)	0.072 (2)	0.0034 (18)	0.0226 (19)	0.0092 (17)
C11	0.093 (3)	0.075 (2)	0.0546 (18)	0.013 (2)	0.015 (2)	−0.0035 (17)
C12	0.081 (3)	0.078 (2)	0.0577 (18)	0.0099 (19)	−0.0069 (19)	−0.0030 (18)
C13	0.0591 (19)	0.076 (2)	0.0605 (18)	0.0006 (18)	−0.0069 (16)	−0.0013 (17)
C14	0.0525 (17)	0.0614 (17)	0.0499 (15)	0.0047 (14)	0.0009 (13)	0.0068 (13)
C15	0.0502 (16)	0.078 (2)	0.0550 (16)	−0.0031 (17)	0.0003 (14)	−0.0035 (16)

### Geometric parameters (Å, °)

**Table:**

Cl—O4	1.415 (4)	C4—C5	1.389 (5)
Cl—O3	1.421 (4)	C4—H4A	0.9300
Cl—O4A	1.423 (5)	C5—C6	1.520 (5)
Cl—O1B	1.424 (5)	C6—C7	1.521 (5)
Cl—O1A	1.424 (5)	C6—H6A	0.9700
Cl—O1C	1.424 (5)	C6—H6B	0.9700
Cl—O3B	1.424 (5)	C7—H7A	0.9700
Cl—O4B	1.427 (5)	C7—H7B	0.9700
Cl—O3C	1.428 (5)	C8—C9	1.503 (5)
Cl—O2C	1.429 (5)	C8—H8A	0.9700
Cl—O3A	1.429 (5)	C8—H8B	0.9700
Cl—O4C	1.431 (5)	C9—C14	1.383 (5)
N1—C1	1.337 (4)	C9—C10	1.392 (4)
N1—C5	1.338 (4)	C10—C11	1.385 (5)
N2—C7	1.495 (4)	C10—H10A	0.9300
N2—C15	1.510 (4)	C11—C12	1.357 (6)
N2—C8	1.512 (4)	C11—H11A	0.9300
N2—H2B	0.9100	C12—C13	1.390 (5)
C1—C2	1.385 (6)	C12—H12A	0.9300
C1—H1A	0.9300	C13—C14	1.382 (5)
C2—C3	1.374 (6)	C13—H13A	0.9300
C2—H2A	0.9300	C14—C15	1.508 (4)
C3—C4	1.373 (6)	C15—H15A	0.9700
C3—H3A	0.9300	C15—H15B	0.9700
			
O4—Cl—O3	111.0 (4)	C7—C6—H6B	108.6
O4A—Cl—O1A	110.1 (5)	H6A—C6—H6B	107.6
O1B—Cl—O3B	110.0 (5)	N2—C7—C6	112.5 (3)
O1B—Cl—O4B	109.8 (5)	N2—C7—H7A	109.1
O3B—Cl—O4B	109.7 (5)	C6—C7—H7A	109.1
O1C—Cl—O3C	109.7 (4)	N2—C7—H7B	109.1
O1C—Cl—O2C	109.6 (4)	C6—C7—H7B	109.1
O3C—Cl—O2C	109.3 (4)	H7A—C7—H7B	107.8
O4A—Cl—O3A	109.5 (4)	C9—C8—N2	102.8 (2)
O1A—Cl—O3A	109.8 (4)	C9—C8—H8A	111.2
O1C—Cl—O4C	109.7 (4)	N2—C8—H8A	111.2
O3C—Cl—O4C	109.4 (4)	C9—C8—H8B	111.2
O2C—Cl—O4C	109.1 (4)	N2—C8—H8B	111.2
C1—N1—C5	118.4 (3)	H8A—C8—H8B	109.1
C7—N2—C15	114.5 (3)	C14—C9—C10	120.1 (3)
C7—N2—C8	112.9 (2)	C14—C9—C8	110.7 (3)
C15—N2—C8	107.5 (2)	C10—C9—C8	129.2 (3)
C7—N2—H2B	107.2	C11—C10—C9	118.3 (4)
C15—N2—H2B	107.2	C11—C10—H10A	120.9
C8—N2—H2B	107.2	C9—C10—H10A	120.9
N1—C1—C2	122.4 (4)	C12—C11—C10	121.3 (4)
N1—C1—H1A	118.8	C12—C11—H11A	119.3
C2—C1—H1A	118.8	C10—C11—H11A	119.3
C3—C2—C1	119.0 (4)	C11—C12—C13	121.0 (4)
C3—C2—H2A	120.5	C11—C12—H12A	119.5
C1—C2—H2A	120.5	C13—C12—H12A	119.5
C4—C3—C2	119.0 (4)	C14—C13—C12	118.1 (4)
C4—C3—H3A	120.5	C14—C13—H13A	120.9
C2—C3—H3A	120.5	C12—C13—H13A	120.9
C3—C4—C5	119.2 (4)	C13—C14—C9	121.0 (3)
C3—C4—H4A	120.4	C13—C14—C15	128.6 (3)
C5—C4—H4A	120.4	C9—C14—C15	110.3 (3)
N1—C5—C4	122.0 (3)	C14—C15—N2	102.8 (3)
N1—C5—C6	115.9 (3)	C14—C15—H15A	111.2
C4—C5—C6	122.1 (3)	N2—C15—H15A	111.2
C5—C6—C7	114.7 (3)	C14—C15—H15B	111.2
C5—C6—H6A	108.6	N2—C15—H15B	111.2
C7—C6—H6A	108.6	H15A—C15—H15B	109.1
C5—C6—H6B	108.6		
			
C5—N1—C1—C2	−2.1 (6)	N2—C8—C9—C10	168.3 (3)
N1—C1—C2—C3	2.5 (6)	C14—C9—C10—C11	0.5 (5)
C1—C2—C3—C4	−1.0 (6)	C8—C9—C10—C11	177.3 (3)
C2—C3—C4—C5	−0.8 (6)	C9—C10—C11—C12	1.4 (6)
C1—N1—C5—C4	0.2 (6)	C10—C11—C12—C13	−1.2 (6)
C1—N1—C5—C6	178.2 (3)	C11—C12—C13—C14	−0.9 (5)
C3—C4—C5—N1	1.2 (6)	C12—C13—C14—C9	2.8 (5)
C3—C4—C5—C6	−176.7 (4)	C12—C13—C14—C15	−177.3 (3)
N1—C5—C6—C7	43.7 (5)	C10—C9—C14—C13	−2.6 (5)
C4—C5—C6—C7	−138.3 (4)	C8—C9—C14—C13	−180.0 (3)
C15—N2—C7—C6	−56.6 (4)	C10—C9—C14—C15	177.5 (3)
C8—N2—C7—C6	179.9 (3)	C8—C9—C14—C15	0.1 (4)
C5—C6—C7—N2	−63.2 (4)	C13—C14—C15—N2	−165.4 (3)
C7—N2—C8—C9	150.7 (3)	C9—C14—C15—N2	14.4 (4)
C15—N2—C8—C9	23.4 (3)	C7—N2—C15—C14	−149.7 (3)
N2—C8—C9—C14	−14.6 (3)	C8—N2—C15—C14	−23.4 (3)

### Hydrogen-bond geometry (Å, °)

**Table:**

*D*—H···*A*	*D*—H	H···*A*	*D*···*A*	*D*—H···*A*
N2—H2B···N1	0.91	2.11	2.769 (4)	129
N2—H2B···O4	0.91	2.39	3.197 (9)	148
N2—H2B···O2A	0.91	2.53	3.222 (19)	133
N2—H2B···O1C	0.91	2.59	3.390 (16)	147
C2—H2A···O1B^i^	0.93	2.30	3.062 (11)	139
C3—H3A···O1A^ii^	0.93	2.62	3.193 (10)	121
C7—H7B···O2A^iii^	0.97	2.50	3.232 (16)	132
C8—H8A···O3	0.97	2.55	3.383 (10)	144
C8—H8A···O2B	0.97	2.39	3.168 (19)	136
C8—H8A···O3C	0.97	2.29	3.189 (17)	154
C8—H8B···O4B^iv^	0.97	2.48	3.167 (17)	128
C11—H11A···O3A^v^	0.93	2.55	3.27 (2)	135
C11—H11A···O3B^v^	0.93	2.57	3.47 (2)	162
C13—H13A···O3^vi^	0.93	2.48	3.299 (9)	148
C13—H13A···O4A^vi^	0.93	2.20	2.976 (8)	140
C13—H13A···O1B^vi^	0.93	2.56	3.44 (2)	158
C13—H13A···O3C^vi^	0.93	2.70	3.444 (17)	138
C15—H15A···O1^iii^	0.97	2.55	3.423 (9)	150
C15—H15B···O2^vi^	0.97	2.48	3.425 (10)	163
C15—H15B···O4A^vi^	0.97	2.55	3.194 (19)	124

Symmetry codes: (i) −*x*−1/2, −*y*+2, *z*−1/2; (ii) *x*+1/2, −*y*+3/2, −*z*+1; (iii) −*x*, *y*−1/2, −*z*+3/2; (iv) −*x*−1, *y*−1/2, −*z*+3/2; (v) −*x*−1/2, −*y*+2, *z*+1/2; (vi) *x*+1, *y*, *z*.

## References

[bb1] Bonnett, R., North, S. A., Newton, R. F. & Scopes, D. I. C. (1983). *Tetrahedron*, **39**, 1401–1405.

[bb2] Bruker (1997). *XSCANS* Bruker AXS Inc., Madison, Wisconsin, USA.

[bb3] Meyers, A. I. & Santiago, B. (1995). *Tetrahedron Lett.* **36**, 5877–5880.

[bb4] North, A. C. T., Phillips, D. C. & Mathews, F. S. (1968). *Acta Cryst.* A**24**, 351–359.

[bb5] Sheldrick, G. M. (2008). *Acta Cryst.* A**64**, 112–122. 10.1107/S0108767307043930 18156677

